# Does Pseudoexfoliation Syndrome Affect the Choroidal Response After Uneventful Phacoemulsification

**DOI:** 10.1167/iovs.61.10.8

**Published:** 2020-08-05

**Authors:** Fatih Aslan, Çağlar Öktem

**Affiliations:** 1 Alaaddin Keykubat University Education and Research Hospital, Department of Ophthalmology, Antalya, Alanya, Turkey; 2 Alaaddin Keykubat University Education and Research Hospital, Antalya, Alanya, Turkey

**Keywords:** choroid, pseudoexfoliation syndrome, phacoemulsification, Haller's layer, optical coherence tomography, SCVL

## Abstract

**Purpose:**

To examine subfoveal and peripapillary choroidal thickness changes after successful phacoemulsification in cataract cases with nonglaucomatous pseudoexfoliation syndrome (PXS).

**Materials and Methods:**

Nineteen cataract patients with PXS and 19 without PXS were included in this prospective and interventional controlled study. Subfoveal and peripapillary choroidal thicknesses were measured before surgery and on the postoperative first day (D1), first week (W1), first month (M1), second month (M2), and third month (M3). Subfoveal choroidal thickness measurement included total subfoveal choroidal thickness (tSFCT), the small choroidal vessel layer (SF-SCVL) thickness, and the large choroidal vessel layer (SF-LCVL) thickness.

**Results:**

The greatest increase in mean tSFCT compared to baseline was observed between W1 and M1 with values of 23.33 ± 2.96 µm and 31.84 ± 2.88 µm, respectively, for the PXS and non-PXS groups (*P* = 0.014). The greatest increase in SF-SCVL thickness compared with baseline occurred at M1 with values of 6.66 ± 1.97 µm and 26.52 ± 1.92 µm, respectively, for the PXS and non-PXS groups (*P* < 0.001). The peripapillary choroidal thickness only showed a significant difference between the groups at the inferior measurement point with values of 117.94 ± 14.15 µm and 137.52 ± 34.53 µm, respectively, for the PXS and non-PXS groups (*P* = 0.032).

**Conclusions:**

Cataract cases with PXS exhibited a different choroidal thickness response compared to non-PXS eyes after successful phacoemulsification. The increased choroidal thickness was particularly observed in Haller's layer in the eyes with PXS and in the choriocapillaris and Sattler's layer in the eyes without PXS.

Pseudoexfoliation syndrome (PXS) is an age-related genetic disorder characterized by the accumulation of extracellular fibrillar material in the entire body, including the eye.^1^ The accumulation of fibrillar material is observed in particular in the anterior surface of the lens and the pupillary margin. The presence of fibrillar material has also been shown to exist in other ocular tissues as well as in extraocular tissues, such as the heart, the lung, the liver, and in the vascular wall.^2^ Pseudoexfoliative glaucoma (PXG) develops as a result of progressive accumulation of pseudoexfoliative material (PXM) in the aqueous humor outflow pathways, together with pigment.

In addition to the anterior segment of the eyes, PXM has been shown in posterior segment tissues such as the posterior ciliary arteries, the vortex veins, and the central retinal artery.^3^ The progressive accumulation in the blood vessels leads to tissue hypoxia and the presence of PXS increases the risk of glaucoma development approximately twofold, in cases of ocular hypertension.^4^

The effect of cataract surgery on retinal and choroidal thickness has been investigated in several previous studies. However, it has been difficult to interpret the results because of the presence of several variables, such as the cataract thickness in the eyes undergoing surgery, the topical agents used as premedication, operative parameters, length of surgery, effective phaco energy (EPE), the amount of intraocular irrigation fluid used, and the postoperative drugs used (with or without nonsteroid anti-inflammatory drops).

We have not come across any previous studies on the peripapillary and subfoveal choroidal thickness changes after cataract surgery, in nonglaucomatous patients with PXS. Thus the purpose of the present study was to investigate the changes in choroidal thickness parameters, following phacoemulsification surgery in unilateral/bilateral PXS without glaucoma. Our hypothesis in planning this study was that the accumulation of PXM restricted vascular expansion. We hypothesized that a different vascular response to surgical stress may therefore emerge in the retinal and choroidal circulation in these eyes due to the accumulated PXM, compared with non-PXS controls.

## Material and Method

### Study Design

The 19 eyes of 19 PXS patients presenting between August 2018 and May 2019 to the Alanya Training and Research Hospital Eye Clinic in Antalya, Turkey, were included in this prospective, interventional, controlled study, together with the 19 eyes of 19 age and sex-matched individuals, as the non-PXS group. Approval for the study was granted by the Alaaddin Keykubat University School of Medicine's Ethics Committee (7-6/2019) and the study was conducted in line with the principles of the Declaration of Helsinki. All participants were given detailed information about the study and provided signed, informed consent forms.

### Patient Selection

The patient group consisted of 19 consecutive patients and only immature cataracts were included, to permit optical coherence tomography (OCT) of sufficient quality (signal level ≥45). The first operated eye was included in the study in case patients with bilateral cataracts were encountered.

All participants underwent a full ophthalmological examination including a best corrected visual acuity (BCVA) measurement, biomicroscopic examination, intraocular pressure measurement using Goldmann applanation tonometry, gonioscopy, fundus examination, central corneal thickness (Tonopachy NT-530P; Nidek, San Jose, CA, USA) measurement, visual field (VF) tests (Octopus; Haag Streit, Mason, OH, USA; 24-2 Swedish interactive threshold algorithm), axial length measurement (Axl) (Eye Cubed; Ellex, Adelaide, Australia), spectral domain optical coherence tomography (SD-OCT) (RTVue-XR Avanti, Optovue Inc., Fremont, CA, USA), and retinal and choroidal evaluation before surgery.

The Lens Opacities Classification System (LOCS) III grading score was used.^5^ Preoperative LOCS scores were evaluated by one of the authors (FA) during a biomicroscopic examination, using high-level illumination and without a light filter.

Patients with a best corrected visual acuity lower than 20/40, a refractive error < ± 3 D, intraocular pressure (IOP) < 21 mm Hg, normal visual field tests, and false-positive and false-negative error rates less than 15%, were included in the study. The inclusion criteria for the cataract patients with PXS additionally included the presence of PXM on the pupillary margin or the anterior lens capsule on dilated fundus examination, a corrected IOP < 21 mm Hg, no previous history of IOP elevation, no previous history of antiglaucomatous drug use, compatibility with normative data on peripapillary nerve fiber analysis (RNFL) with OCT and ganglion cell complex (GCC) evaluation, a visual field test mean deviation and pattern deviation value within the 95% confidence interval, and the absence of cupping, notching or pallor on funduscopic examination of the optic nerve head. Patients requiring iris manipulation or a capsular tension ring during surgery were excluded.

### Cataract Surgery

Cataract surgery was performed under topical anesthesia (proparacaine hydrochloride, Alcaine; Alcon Labs Inc., Geneva, Switzerland) by an experienced surgeon (FA). Coaxial phacoemulsification was performed using a Kelman 30° 0.9-mm microtip with the quick chop technique on the Infiniti Vision System (Alcon Lab Inc), on all patients in this study. A foldable intraocular lens (Acrysoft SA60AT; Alcon Labs Inc.) was inserted inside the capsule at the end of surgery in all cases and the same viscoelastic material (DisCoVisc; Alcon Lab Inc.) was used for each patient. Intraocular lens power, effective phaco time, duration of surgery, and the amount of intraocular irrigation fluid used, were noted at the end of surgery. Finally, 1 mg/0.1 mL cefuroxime axetil was administered into the anterior chamber, through the corneal side-entry for endophthalmitis prophylaxis, at the end of surgery.

Postoperative follow-up examinations were performed on the first day (D1), the first week (W1), the first month (M1), the second month (M2), and the third month (M3). Patients were started on one drop moxifloxacin 0.5% (Vigamox; Alcon Labs Inc.) every 4 h, and one drop prednisolone acetate 1% (Pred Forte; Allergan, Dublin, Ireland) every four hours for three weeks.

### Optical Coherence Tomography Imaging

**Figure 1.**
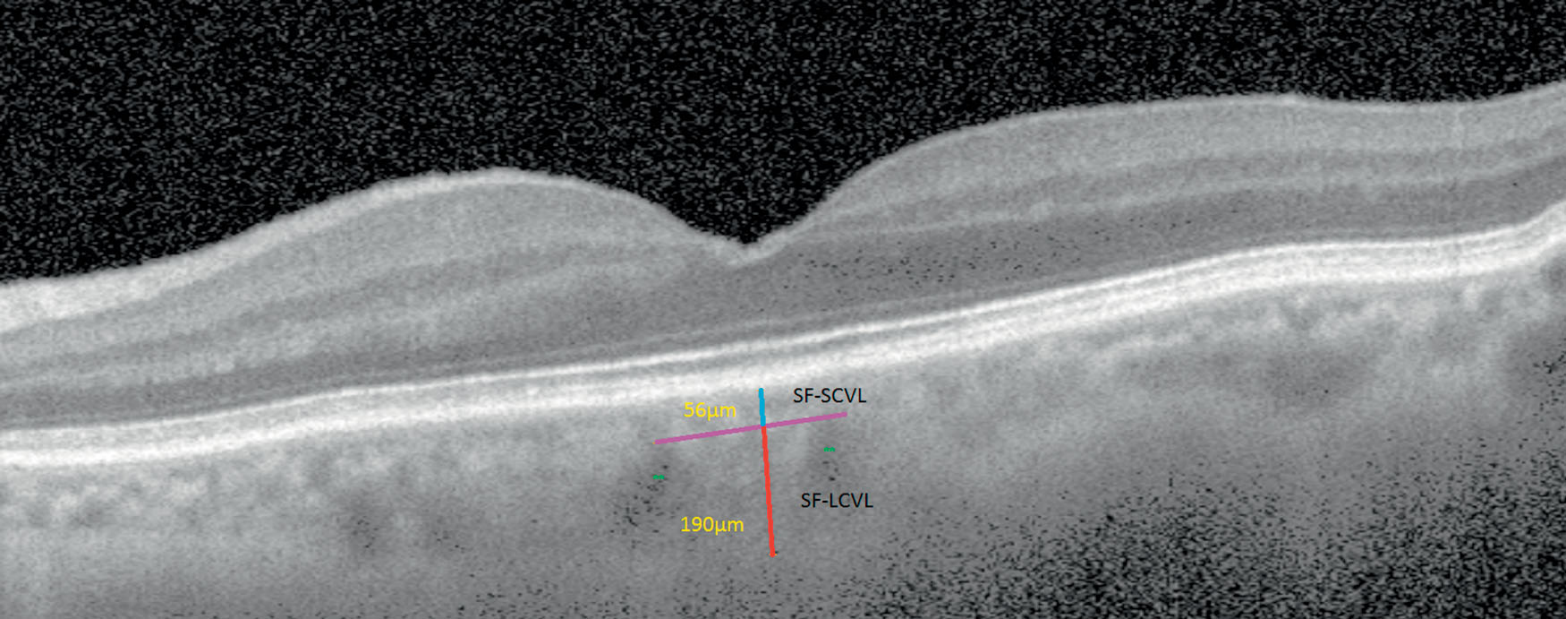
SFCT measurements. Large choroidal vessel layer thickness (Haller's layer) (SF-LCVL) was measured from the inner border of the choroid-scleral junction to the innermost point of the large choroidal vessel at subfoveal location (*red line*). *Double asterisks* indicate large choroidal vessels. Sattler's layer/choriocapillaris layer (SF-SCVL) is the distance from the outer edge of the hyper-reflective retinal pigment epithelium to the *red line* and is indicated by the *turquoise-colored line*. Sattler's layer/choriocapillaris layer (SF-SCVL) is separated from the Haller's layer by a *purple line*. Sum of SF-SCVL and SF-LCVL layers gives total subfoveal thickness. All measurements were performed using the RTVue measurement tool.

All study eyes were imaged with the RTVue OCT (Optovue Inc., Fremont, CA, USA), a high-speed and high-resolution SD-OCT device with a central wavelength of 840 nm, scan rate of 26,000 A-scans/s, and axial resolution of 5 µm. For the purpose of this study, horizontal B-scan images centered on the fovea were used to perform computed tomography (CT) measurements. Each B-scan image is constructed from a number of line scans through the same retinal locations, and each line scan consists of 1024 A-scans. With all SD-OCT systems, the signal return from an object is higher when the object is placed nearer the zero delay. The RTVue software provides a “Chorioretinal” acquisition mode, which moves the zero delay toward the bottom of the screen, thereby enhancing the signal returning from the choroidal region which is located in the lower part of the screen. The choroid-sclera interface was defined as the outermost dark-to-bright boundary. Bias was eliminated by masking patient data during the measurements and the choroidal thickness measurements were performed manually by two masked physicians (FA, ÇÖ). Measurement of the large choroidal vessel layer thickness (LCVL) and the Sattler's layer/choriocapillaris thickness (SCVL) at the subfoveal location was undertaken using the method suggested by Branchini et al.^6^ (Fig. 1). A large choroidal vessel was defined as a lumen of at least 100 µm in diameter within the choroid. Choroidal thickness was measured perpendicularly from Bruch's membrane, equivalent to the choroid-sclera interface at the fovea, and at two more points located at 1000 µm nasal to the fovea and 1000 µm temporal to the fovea, using the RTVue software's “chorioretinal imaging mode.” All basal OCT scans were performed at the same time of the day (9 to 11 am) to avoid diurnal fluctuations. Three readings were taken by each grader at different times, and each set of readings was averaged and recorded by the two graders themselves. The interobserver reproducibility of the choroidal measurements was evaluated by measuring the interclass correlation coefficient (ICC).

### Statistical Analysis

All statistical procedures were performed using the SPSS 22 (IBM, 2013) software. The sample analysis was conducted before the study. We used the G*Power (Faul, Erdfelder, Lang, and Buchner, 2007) (unpublished observations) software to calculate the sample size. The analysis of variance for repeated measurements used an effect size of 0.20, error of 5%, and confidence interval of 95%. The minimum sample size was calculated as 28 for two groups and 12 measurements. “Box plots” were used to show the distribution of the data. The Bland-Altman analysis and t-tests were used to report on the inter-observer repeatability. The significance level was set at 95% with *P*-value of ≤ 0.05. The *t* test was used to determine equivalence between the PXS and non-PXS groups before the procedure and the presence of any difference between the groups, in terms of the measurements. Cohen's d formula was calculated online to determine whether the differences identified with the *t* test were significant. The post hoc Tukey test was applied to determine the existence of any difference between the measurements.

## Results

Age, sex, BCVA, cataract hardness, CTT (Central corneal thickness), AxL, VF-MD (Visual field-mean deviation), RNFL, GCC, duration of surgery, EPE, and IOP values were similar between the two groups (*P* > 0.05 for all). ICCs were calculated to assess intragrader and intergrader reliability between the two observers. Intragrader ICCs of grader 1 for preoperative, D1, W1, M1, M2, and M3 readings were 0.977, 0.990, 0.980, 0.978, 0.969, and 0.991, and those of grader 2 were 0.969, 0.984, 0.967, 0.982, 0.985, and 0.977, respectively. The interreader ICCs for preoperative, D1, W1, M1, M2, and M3 readings were 0.946, 0.991, 0.987, 0.977, 0.990, and 0.992, respectively. The patients’ demographic and clinical characteristics are presented in Table 1. Pseudophakic cystoid macular edema (PCME) was detected at the M1 follow-up in one (5.26%) of the 19 patients in the PXS group, and this patient's data were excluded from the choroid analyses.

**Table 1.** A Comparison of Preoperative and Intraoperative Parameters in the Study and Control Groups

	Group	N	Mean	SD	*t*	*P* Value
Age	PXS+	19	68.316	5.100	1.405	0.169
	PXS−	19	65.263	7.978		
BCVA	PXS+	19	0.858	0.278	0.391	0.698
	PXS−	19	0.825	0.232		
Cataract Grade	PXS+	19	2.579	0.607	1.708	0.288
	PXS−	19	2.368	0.597		
IOL power (D)	PXS+	19	20.5	2.48	0.908	0.486
	PXS−	19	21.2	3.41		
CCt (µm)	PXS+	19	533.684	18.523	−1.898	0.066
	PXS−	19	550.474	33.815		
AxL (mm)	PXS+	19	23.232	0.605	−0.465	0.645
	PXS−	19	23.359	1.030		
VF MD	PXS+	19	2.016	1.259	−1.009	0.320
	PXS−	19	2.416	1.182		
GCC	PXS+	19	95.421	5.167	−0.314	0.755
	PXS−	19	95.895	4.054		
RNFL	PXS+	19	102.421	8.023	−1.574	0.124
	PXS−	19	106.579	8.255		
Surgery Time (minute)	PXS+	19	6.168	1.760	0.918	0.365
	PXS−	19	5.711	1.274		
EPT (second)	PXS+	19	1.947	1.365	0.753	0.456
	PXS−	19	1.675	0.791		
Irr Fluid (cc)	PXS+	19	31.158	7.939	−0.226	0.823
	PXS−	19	31.684	6.343		
Preop. IOP	PXS+	19	15.211	1.903	0.615	0.542
	PXS−	19	14.789	2.299		

CCt, central corneal thickness; VF MD, visual field mean deviation; EPT, effective phaco time; Irr Fluid, irrigating fluid; IOL. intraocular lens.

Mean preoperative total subfoveal choroidal thickness (tSFCT) values in the PXS and non-PXS groups were 235.55 ± 29.15 µm and 251.00 ± 31.08 µm, respectively (*P* = 0.129). The tSFCT exhibited a rising trend at all follow-up times in both groups. The differences between the groups were statistically significant at W1 and M1 follow-ups (Table 2).

**Table 2.** A Comparison of Changes in **t**SFCT in the PXS and Non-PXS Groups

Time	Group	N	Mean	SD	*P* Value	Cohen's d	Difference 95% CI
Pre.	PXS+	18	235.556	29.157	0.129	−0.512	15.444 [−4.69659, 35.58459]
	PXS−	19	251.000	31.082			
D1	PXS+	18	249.000	30.102	0.442	−0.256	7.842 [−12.62955, 28.31355]
	PXS−	19	256.842	31.174			
W1	PXS+	18	259.167	35.312	**0.047**	−0.676	22.622 [0.27828, 44.96572]
	PXS−	19	281.789	31.615			
M1	PXS+	18	258.889	28.533	**0.020**	−0.804	23.953 [4.0608, 43.8452]
	PXS−	19	282.842	30.931			
M2	PXS+	18	246.500	28.335	0.076	−0.804	17.816 [−1.99444, 37.62644]
	PXS−	19	264.316	30.874			
M3	PXS+	18	244.111	27.766	0.186	−0.444	12.784 [−6.46266, 32.03066]
	PXS−	19	256.895	29.788			

Significant *P* values are in bold.

Pre, preoperative; CI, confidence interval.

The greatest increase in tSFCT compared with baseline was observed between W1 and M1 with 23.33 ± 2.96 µm in the PXS group and 31.84 ± 2.88 µm in the non-PXS group (*P* = 0.014). The mean preoperative subfoveal small choroidal vessel layer (SF-SCVL) thickness was 64.05 ± 17.18 µm in the PXS group and 55.00 ± 10.82 µm in the non-PXS group (*P* = 0.051). A significant difference in SF-SCVL thickness was only determined between the two groups at M1, with values of 71.16 ± 16.74 µm in the PXS group and 81.52 ± 11.98 µm in the non-PXS group (*P* = 0.037). The amount of change in SF-SCVL at M1 compared with baseline values was 6.66 ± 1.97 µm in the PXS group and 26.52 ± 1.92 µm in the non-PXS group (*P* = <0.001).

**Figure 2.**
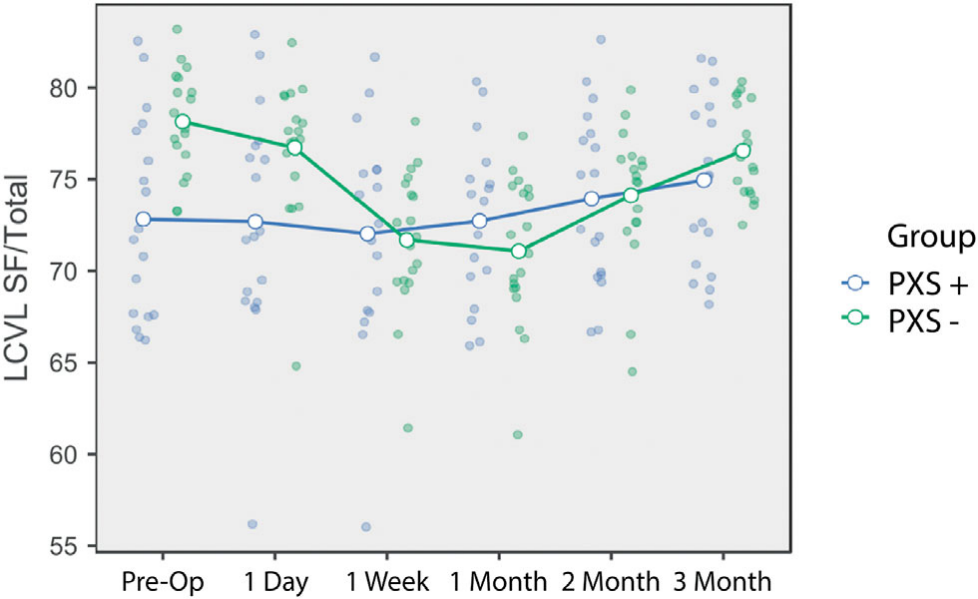
A comparison of the changes in the ratio between the subfoveal LCVL thickness and the total choroidal thickness at baseline and postoperative follow-up.

Preoperative subfoveal large choroidal vessel layer (SF-LCVL) thickness values were 169.94 ± 21.73 µm in the PXS group and 195.94± 23.82 µm in the non-PXS group (*P* = 0.001). Although PXS group SF-LCVL values were lower than those of the non-PXS group at all-time points, a statistically significant difference between the groups was only observed at D1, M1, and M3. Intragroup SF-LCVL analysis revealed a statistically significant increase in the PXS group at all time points after surgery (Fig. 2), although these increases in the non-PXS group were not statistically significant. The greatest increase in the PXS and non-PXS groups was at the M1 follow-up, at 17.77 ± 2.17 µm and 5.36 ± 2.11 µm, respectively (*P* = 0.001).

The preoperative SF-SCVL thickness to tSFCT ratio differed significantly between the PXS and non-PXS groups (27.19% ± 5.39% vs. 21.86% ± 2.77%, respectively; *P* < 0.001). The variation between the groups continued at the D1 check-up but disappeared at other time points (Table 3). The change in this ratio in the PXS group was not significant at other time points but increased statistically significantly in the non-PXS group at W1, M1, and M2. In the non-PXS group, the greatest increase compared to baseline was at the M1 check-up, at 7.07% ± 0.59% (*P* < 0.001).

**Table 3.** A Comparison of the Postoperative Changes in Choroidal Parameters

	PXS +		PXS−		
Time	Mean	SD	Mean	SD	*P* Value
First week					
tSFCT	23.6	11.5	30.8	16.7	0.138
SF-SCVL	9.2	13.1	24.6	9.9	**<0.001**
SF-LCVL	14.4	7.6	6.2	12.9	**0.025**
SCVL/tSFCT	0.8	3.0	6.5	3.2	**<0.001**
N-tCT	12.3	5.2	22.2	13.1	**0.005**
T-tCT	13.3	8.0	20.2	13.1	0.063
First month					
tSFCT	23.3	9.2	31.8	10.6	**0.014**
SF-SCVL	6.7	4.1	26.5	9.3	**<0.001**
SF-LCVL	16.7	8.8	5.3	9.7	**0.001**
SCVL/SFCT	0.1	1.9	7.1	3.4	**<0.001**
N-tCT	17.9	16.3	23.7	13.6	0.246
T-tCT	8.1	22.2	23.4	14.1	**0.017**
Second month					
tSFCT	10.9	8.7	13.3	7.5	0.380
SF-SCVL	0.2	4.4	13.1	8.9	**<0.001**
SF-LCVL	10.7	8.6	0.2	10.6	**0.002**
SCVL/SFCT	−1.1	1.9	4.0	3.6	**<0.001**
N-tCT	6.7	17.7	7.3	11.4	0.895
T-tCT	−0,7	19.2	7.9	14.8	0.317
Third month					
tSFCT	8.6	8.2	5.9	4.4	0.235
SF-SCVL	−2.7	5.2	5.2	4.8	**<0.001**
SF-LCVL	11.2	8.9	0.7	6.3	**<0.001**
SCVL/SFCT	−2.1	2.4	1.6	2.0	**<0.001**
N-tCT	4.4	16.7	1.2	10.8	0.487
T-tCT	−4.3	19.8	0.1	14.1	0.436

Significant *P* values are in bold.

N-tCT, total choroidal thickness 1 mm from the fovea in the nasal direction; T-tCT, total choroidal thickness 1 mm from the fovea in the temporal direction.

Initial CT measurement values at the temporal point 1 mm from the fovea were 234.72 ± 36.65 µm in the PXS group and 233.15 ± 31.20 µm in the non-PXS group (*P* = 0.889). No statistically significant difference was observed between mean values at subsequent time points. In terms of changes after baseline, a significant difference between the groups was observed only at M1, with values of 8.14 ± 22.2 µm in the PXS group and 23.41 ± 14.13 µm in the non-PXS group (*P* = 0.017).

The initial CT measurement values 1 mm nasal to the fovea were 208.83 ± 28.53 µm in the PXS group and 211 ± 31.45 µm in the non-PXS group (*P* = 0.828). The amount of change from baseline at W1 was 12.32 ± 5.24 µm in the PXS group and 22.21 ± 13.41 µm in the non-PXS group (*P* = 0.005). The greatest increase in thickness compared to baseline values occurred at M1, with values of 17.88 ± 2.96 µm in the PXS group and 23.68 ± 2.88 µm in the non-PXS group (*P* = 0.246). Decreasing trends were observed in both groups after the first month, and preoperative values were again observed at M3.

The peripapillary choroidal thickness (PPCT) analysis revealed no statistically significant difference between the initial superior, temporal and nasal measurements at any postoperative time in or between the groups. Preoperative inferior PPCT was 117.94 ± 14.15 µm in the PXS group and 137.52 ± 34.53 µm in the non-PXS group (*P* = 0.032). This statistically significant difference between the groups persisted at all-time points, including M3 (Table 4). Inferior PPCT values at M1 increased by 4.61 ± 2.19 µm in the PXS group and 9.84± 2.12 µm in the non-PXS group compared with baseline levels (*P* = 0.228). The change in IOP compared with the baseline values was 0.33 ± 0.35 mm Hg in the PXS group and −0.10 ± 0.34 mm Hg in the non-PXS group (*P* = 0.172).

**Table 4.** A Comparison of Changes in Peripapillary Choroidal Thickness

		PXS (+)		PXS (−)			
	Time	Mean ± SD		Mean ± SD		*P* Value	Cohen's d
**PPCT-T**	*Pre.*	153.83	27.16	161.21	41.87	0.532	−0.208
	*D1*	153.44	25.19	164.26	39.87	0.334	−0.322
	*W1*	159.88	23.62	175.52	51.71	0.249	−0.385
	*M1*	161.00	24.18	175.84	51.41	0.273	−0.366
	*M2*	156.38	23.37	170.21	51.56	0.306	−0.342
	*M3*	154.83	22.17	167.89	51.36	0.327	−0.327
**PPCT-S**	*Pre.*	164.82	24.15	180.85	43.25	0.178	−0.452
	*D1*	167.41	23.38	182.33	41.57	0.192	−0.438
	*W1*	172.93	22.90	186.39	43.87	0.255	−0.381
	*M1*	173.08	22.98	187.94	43.36	0.204	−0.426
	*M2*	168.87	22.48	182.47	42.49	0.204	−0.426
	*M3*	166.45	20.79	179.71	43.00	0.238	−0.395
**PPCT-N**	*Pre.*	150.66	25.59	148.84	34.88	0.858	0.059
	*D1*	153.27	24.85	151.05	33.69	0.821	0.075
	*W1*	158.77	24.01	160.26	47.24	0.906	−0.039
	*M1*	160.55	23.74	162.63	47.25	0.868	−0.055
	*M2*	155.16	23.57	157.36	44.33	0.853	−0.062
	*M3*	153.77	24.54	155.89	45.07	0.861	−0.058
**PPCT-I**	*Pre.*	117.94	14.15	137.52	34.53	**0.032**	−0.735
	*D1*	119.72	14.05	139.73	33.43	**0.025**	−0.773
	*W1*	122.83	14.12	145.89	39.22	**0.024**	−0.774
	*M1*	122.55	14.65	147.36	38.51	**0.015**	−0.843
	*M2*	116.38	13.47	142.52	38.43	**0.010**	−0.898
	*M3*	113.27	12.90	140.05	38.55	**0.008**	−0.921

Significant *P* values are in bold.

PPCT-T, peripapillary choroidal thickness-temporal; PPCT-S, peripapillary choroidal thickness-superior; PPCT-N, peripapillary choroidal thickness-nasal; PPCT-I, peripapillary choroidal thickness-inferior; Pre, preoperative; D7, postoperative seventh day.

## Discussion

Studies concerning the biochemical effects of cataract surgery on the retina and choroid are yielding new information. One of the most important findings is that cataract surgery increases proinflammatory gene expression and protein secretion, in the retinal and choroidal layers.^7^ A study on rhesus monkeys has revealed that cataract surgery increased the macular thickness and impaired the outer blood-retina barrier.^8^

Choroidal and retinal thickness changes after successful phacoemulsification are the subject of considerable research. However, we are not aware of any previous study on the changes in subfoveal and peripapillary choroidal thickness after successful phacoemulsification in cataract cases with PXS.

The most important finding from this study is that the initial and early postoperative period change in total subfoveal choroidal thickness in cataract cases with PXS, differed significantly from the non-PXS controls. The increase in choroidal thickness as a response to cataract surgery was mostly associated with expansion of the Haller's layer in cases with PXS, and of the choriocapillaris and Sattler's layer in non-PXS controls. The lower amount of expansion at the first week and the first month in the choriocapillaris and small-diameter choroidal vessel layer in cases with PXS supports the hypothesis that PXM first appears with impairment of the small vessel diameter.

Another interesting study finding is that tSFCT values in the cataract group with PXS were still significantly higher, compared with baseline at M3 check-ups. Although tSFCT was also higher at M3 compared with initial values in the non-PXS group, the difference there was not statistically significant.

The choroid consists of vascular structures and the surrounding stromal tissue. We detected statistically significant thickening in the subfoveal choroidal layer after phacoemulsification in both groups. However, whether this change is associated with expansion of the vascular or extravascular stromal structures is unknown.

Our study results showed no significant change after surgery in the SCVL to tSFCT ratio in eyes with PXS, whereas this ratio exhibited significant change in the non-PXS controls, particularly in the first two months. This may be due to restriction of the expansive property of tissue with PXM accumulation in the stromal area, in addition to the vascular structures.

Yılmaz et al.^9^ followed up 65 patients who had undergone successful phacoemulsification for a period of 12 months and reported a continuous, albeit statistically insignificant, increase in SFCT. Our findings are not consistent with that study, because we observed a decreasing trend in SFCT values from the first month onward in both the PXS and non-PXS groups. We think that this inconsistency may be due to the variation in IOP, because we observed no significant variation in IOP in either group compared with baseline, over the three-month follow-up period. Researchers reported a 4-mm Hg decrease from baseline mean IOP values over one year in their patients, whereas we observed a change of 0.35 mm Hg at the end of three months.

We detected PCME in only one patient (5.26%) in the PXS group. This patient's intraoperative characteristics were similar to those of the other PXS group members. No systemic or ocular risk factor for PCME was present; however, the subfoveal SCVL thickness to total subfoveal choroidal thickness ratio in the initial period was 40.37% in this patient, compared with a mean value of 27.19% in the other PXS patients. A relatively high SCVL/tSFCT ratio before phacoemulsification in PXS patients may perhaps represent a risk factor for PCME.

The incidence of PCME is approximately 1% after successful cataract surgery in cases with no risk factors (diabetes, uveitis, epiretinal membrane, retinal vein occlusion, or intraoperative capsule rupture), although PCME is associated with impairment of the blood-aqueous barrier after intraocular surgery.^10^ Cases with PXS have been linked to more severe and more prolonged flare because of fragility of the blood-aqueous barrier after successful cataract surgery.^11^ Although these patients are known to exhibit a more severe inflammatory response after cataract surgery, there is no consensus on the optimal postoperative follow-up protocol. In agreement with the present research, a recent study from Finland reported a 4% rate of PCME after successful cataract surgery in eyes with PXS, that were treated after surgery with steroid monotherapy only.^12^ These supportive findings suggest that PXS may be an independent risk factor for PCME.

Insufficient choroidal blood flow is implicated as a risk factor in the progression of glaucoma, independently of IOP.^13^^,^^14^ The “Early Manifest Glaucoma Trial” has described the presence of PXS as the most important risk factor in glaucomatous progression.^15^ Studies assessing choroidal thickness using OCT in PXS cases have generally reported a thinner choroid compared with non-PXS eyes.^16^^–^^20^ The PPCT values of the PXS cases constituting our PXS group were significantly thinner than those of the non-PXS group, but only in the inferior quadrant. No difference was observed in the increased PPCT thickness response in our case series with PXS compared with the non-PXS controls. However, the lower PPCT inferior thickness suggests that the inferior location may be the first to be affected because of gravity during the process of PXM accumulation. Dursun et al.^21^ have compared the PPCT in cases with PXS and PXG to healthy control subjects and found thinner PPCT values in the patient group in all quadrants other than the superior one. This result supports the speculation in our article that gravity plays a role.

Although tSFCT values at baseline exhibited no statistically significant difference between the two groups in the present study, subfoveal LCVL thickness was significantly lower in our PXS groups than in the non-PXS controls. In addition, optical coherence tomography angiography (OCTA) examinations of cases with PXS have been reported to show greater expansion, in both the superficial vascular plexus and the deep vascular plexus in the foveal avascular zone, compared with the non-PXS controls.^22^ Sarrafpour et al.^23^ measured the subfoveal vessel diameters of eyes with PXS using SD-OCT and reported that the mean choroidal vessel diameter in the subfoveal area was 13 µm smaller in unilateral PXS cases than in the contralateral eye. These findings demonstrate the effects of the degeneration caused by PXM in the retinal and choroidal vessels.

After Spaide et al.'s^24^ semiquantitative evaluation of the choroid using EDI-OCT, various studies on choroidal thickness in various ocular and systemic disorders have been published.^25^^,^^26^ Examination of the choroid using SCVL and LCVL has recently become a popular method for demonstrating the effect of systemic and ocular disorder on the choroid.^27^^–^^29^ An important aspect of the current study on choroidal thickness changes after successful phacoemulsification is the measurement of the choroidal thickness in two different sections, including the choriocapillaris-Sattler's layer (SCVL) and the Haller's layer (LCVL).

The limitations of the present study include the limited nature of the study group, the manual OCT measurements, and the short follow-up period. The potential magnification effect of cataract surgery on the OCT image because of the variable optic system should be taken into account. The most significant aspect of our study is that it is the first to assess the effect of PXS on choroidal changes after successful phacoemulsification, through separate examination of the different layers. Another notable aspect of this study includes the fact that all surgery was performed by a single surgeon using the same device and technique and with the same postoperative follow-up protocol.

In conclusion, this study has revealed that the change in choroidal thickness after successful phacoemulsification in eyes with PXS differed from that in non-PXS eyes. The increase in postoperative thickness between the first week and the first month in the eyes with PXS was particularly associated with Haller's layer, whereas the choroidal expansion in the non-PXS eyes was mostly related to the choriocapillaris and Sattler's layer. We think that this may be due to the microangiopathic effect of pseudoexfoliative material accumulating in the choroid in eyes with PXS in which glaucoma has not yet developed. These results now need to be supported with OCTA and aqueous flare meter results in larger patient groups. Our study elicited new information concerning the effect of PXS on the choroidal vessels, and we believe that it will act as a useful guide for future research.
